# Ovarian cancer population screening and mortality after long-term follow-up in the UK Collaborative Trial of Ovarian Cancer Screening (UKCTOCS): a randomised controlled trial

**DOI:** 10.1016/S0140-6736(21)00731-5

**Published:** 2021-06-05

**Authors:** Usha Menon, Aleksandra Gentry-Maharaj, Matthew Burnell, Naveena Singh, Andy Ryan, Chloe Karpinskyj, Giulia Carlino, Julie Taylor, Susan K Massingham, Maria Raikou, Jatinderpal K Kalsi, Robert Woolas, Ranjit Manchanda, Rupali Arora, Laura Casey, Anne Dawnay, Stephen Dobbs, Simon Leeson, Tim Mould, Mourad W Seif, Aarti Sharma, Karin Williamson, Yiling Liu, Lesley Fallowfield, Alistair J McGuire, Stuart Campbell, Steven J Skates, Ian J Jacobs, Mahesh Parmar

**Affiliations:** a MRC Clinical Trials Unit, Institute of Clinical Trials and Methodology, University College London, London, UK; b Clinical Epidemiology, Institute of Health Informatics, University College London, London, UK; c Department of Women's Cancer, Institute for Women's Health, University College London, London, UK; d Department of Cellular Pathology, Barts Health NHS Trust, London, UK; e Department of Clinical Biochemistry, Barts Health NHS Trust, London, UK; f Department of Gynaecological Oncology, Barts Health NHS Trust, London, UK; g Department of Health Policy, London School of Economics and Political Science, London, UK; h Department of Economics, University of Piraeus, Athens, Greece; i Department of Gynaecological Oncology, Queen Alexandra Hospital, Portsmouth, UK; j Wolfson Institute of Preventive Medicine, CRUK Barts Cancer Centre, Queen Mary University of London, London, UK; k Department of Cellular Pathology, University College London Hospitals NHS Trust, London, UK; l Department of Gynaecological Oncology, University College London Hospitals NHS Trust, London, UK; m Department of Gynaecological Oncology, Belfast City Hospital, Belfast, UK; n Department of Obstetrics and Gynaecology, Ysbyty Gwynedd, Bangor, UK; o Division of Gynaecology and Cancer Sciences, St Mary's Hospital, Manchester, UK; p Department of Obstetrics and Gynaecology, University Hospital of Wales, Cardiff, UK; q Department of Gynaecological Oncology, Nottingham City Hospital, Nottingham, UK; r MGH Biostatistics, Department of Medicine, Massachusetts General Hospital, Boston, MA, USA; s Department of Medicine, Harvard Medical School, Boston, MA, USA; t Sussex Health Outcomes Research and Education in Cancer, SHORE-C, Brighton and Sussex Medical School, University of Sussex, Brighton, UK; u Create Health, London, UK; v Department of Women's Health, University of New South Wales, Sydney, NSW, Australia

## Abstract

**Background:**

Ovarian cancer continues to have a poor prognosis with the majority of women diagnosed with advanced disease. Therefore, we undertook the UK Collaborative Trial of Ovarian Cancer Screening (UKCTOCS) to determine if population screening can reduce deaths due to the disease. We report on ovarian cancer mortality after long-term follow-up in UKCTOCS.

**Methods:**

In this randomised controlled trial, postmenopausal women aged 50–74 years were recruited from 13 centres in National Health Service trusts in England, Wales, and Northern Ireland. Exclusion criteria were bilateral oophorectomy, previous ovarian or active non-ovarian malignancy, or increased familial ovarian cancer risk. The trial management system confirmed eligibility and randomly allocated participants in blocks of 32 using computer generated random numbers to annual multimodal screening (MMS), annual transvaginal ultrasound screening (USS), or no screening, in a 1:1:2 ratio. Follow-up was through national registries. The primary outcome was death due to ovarian or tubal cancer (WHO 2014 criteria) by June 30, 2020. Analyses were by intention to screen, comparing MMS and USS separately with no screening using the versatile test. Investigators and participants were aware of screening type, whereas the outcomes review committee were masked to randomisation group. This study is registered with ISRCTN, 22488978, and ClinicalTrials.gov, NCT00058032.

**Findings:**

Between April 17, 2001, and Sept 29, 2005, of 1 243 282 women invited, 202 638 were recruited and randomly assigned, and 202 562 were included in the analysis: 50 625 (25·0%) in the MMS group, 50 623 (25·0%) in the USS group, and 101 314 (50·0%) in the no screening group. At a median follow-up of 16·3 years (IQR 15·1–17·3), 2055 women were diagnosed with tubal or ovarian cancer: 522 (1·0%) of 50 625 in the MMS group, 517 (1·0%) of 50 623 in the USS group, and 1016 (1·0%) of 101 314 in the no screening group. Compared with no screening, there was a 47·2% (95% CI 19·7 to 81·1) increase in stage I and 24·5% (−41·8 to –2·0) decrease in stage IV disease incidence in the MMS group. Overall the incidence of stage I or II disease was 39·2% (95% CI 16·1 to 66·9) higher in the MMS group than in the no screening group, whereas the incidence of stage III or IV disease was 10·2% (−21·3 to 2·4) lower. 1206 women died of the disease: 296 (0·6%) of 50 625 in the MMS group, 291 (0·6%) of 50 623 in the USS group, and 619 (0·6%) of 101 314 in the no screening group. No significant reduction in ovarian and tubal cancer deaths was observed in the MMS (p=0·58) or USS (p=0·36) groups compared with the no screening group.

**Interpretation:**

The reduction in stage III or IV disease incidence in the MMS group was not sufficient to translate into lives saved, illustrating the importance of specifying cancer mortality as the primary outcome in screening trials. Given that screening did not significantly reduce ovarian and tubal cancer deaths, general population screening cannot be recommended.

**Funding:**

National Institute for Health Research, Cancer Research UK, and The Eve Appeal.

## Introduction

Ovarian cancer remains the most deadly of all gynaecological cancers. Most patients (58%) are diagnosed at an advanced stage (III or IV), which is associated with poor survival (5-year survival is 27% for stage III and 13% for stage IV ovarian cancer).^1^ The greater than 90% survival rates in women detected at stage I^1^ has spurred international efforts in early detection, spanning across four decades.^2^, ^3^, ^4^, ^5^, ^6^ All trials have used combinations of the biomarker CA125 and pelvic imaging using transvaginal ultrasound scans (TVS). Despite these extensive endeavours, to date there is no evidence that screening for ovarian cancer saves lives.^7^, ^8^, ^9^

In our multicentre randomised trial (UK Collaborative Trial of Ovarian Cancer Screening [UKCTOCS]), 202 638 women from the general population were randomly assigned to two annual screening groups—multimodal screening (MMS; longitudinal CA125 and second line TVS) and ultrasound screening (USS; TVS first and second-line test), and a no screening group. We reported previously (median follow-up of 11·1 years), that an absolute proportion of 13% more women with ovarian, tubal, and peritoneal cancer were diagnosed with stage I or II disease in the MMS group than in the no screening group. There was no change in stage in the USS group. There was no evidence of a reduction in disease-specific deaths in either screened group compared with the no screening group using the Cox version of the log-rank test. The observed reduction in deaths was delayed and the cumulative mortality curves appeared to be diverging at the time of previous reporting.^9^ Therefore, we aimed to continue follow-up and report here on the long-term mortality effects of ovarian cancer screening in UKCTOCS.

**Research in context**

**Evidence before this study**

We searched PubMed from Jan 1, 2015, to Dec 31, 2020, with no language restrictions for randomised controlled trials for ovarian cancer screening that reported mortality data. The following keywords were used to search the database: “ovarian cancer” AND “randomised controlled trial” AND “screening” AND “mortality”. We found two relevant publications. In the UK Collaborative Trial of Ovarian Cancer Screening (UKCTOCS; n=202 638), at a median follow-up of 11·1 years, no significant reduction in deaths from ovarian cancer was seen in either of the screen groups (multimodal or ultrasound) compared with the no screening group. A reduction in deaths was seen but was delayed and only apparent after about 7 years. There was a suggestion that 15% fewer women in the multimodal screening group and 11% fewer in the ultrasound screening group died from ovarian cancer compared with the no screening group. Additionally, a significantly greater proportion (13%) of women with ovarian cancers in the multimodal group but not in the ultrasound group were found at an earlier stage (stage I and II) compared with the no screening group. As the data did not definitively answer the question of whether screening saved lives, follow-up was continued to gather more evidence. The Ovarian Cancer Screening arm of the Prostate Lung Colorectal Ovarian (PLCO) cancer trial in the USA is the only other large randomised controlled trial (n=78 216) to explore mortality benefit. Following extended follow-up (median 14·7 years), the trial confirmed previous findings of no ovarian cancer mortality reduction between the screen and control arms.

**Added value of this study**

Long-term follow-up (median follow-up >16 years after recruitment) in the largest ovarian cancer screening trial, to our knowledge, provides definitive new evidence that neither screening approaches used in UKCTOCS reduced deaths from ovarian cancer, compared with no screening. This result was despite a 47·2% increase in incidence of women with ovarian and tubal cancer diagnosed at stage I and 24·5% decrease in those diagnosed with stage IV disease in the multimodal group compared with the no screening group. Importantly, however, there was only a 10·2% decrease in overall incidence of stage III or IV disease.

**Implications of all the available evidence**

General population screening for ovarian and tubal cancer with either approach used in UKCTOCS cannot currently be recommended. We need a screening strategy that can detect ovarian and tubal cancer in asymptomatic women even earlier in its course and in a larger proportion of women than the tests used in the trial. Meanwhile, our results emphasise the importance of having ovarian and tubal cancer mortality as the primary outcome in screening trials.

## Methods

### Study design and participants

We did a randomised controlled trial (UKCTOCS) of postmenopausal women aged 50–74 years from the general population recruited through 13 centres in National Health Service (NHS) Trusts in England, Wales, and Northern Ireland with use of age-sex registers of 27 primary care trusts.^10^

We commissioned specialised software from the NHS to randomly select women aged 50–74 years and then flag them on primary care trusts' registers and allow electronic transfer of their personal and general practice details. We then sent women personal invitations and logged replies on the trial management system. Women attended a recruitment clinic at the regional centre where they viewed an information video, completed a recruitment questionnaire, and provided written consent and a baseline serum sample. We scanned recruitment questionnaires at the coordinating centre into a bespoke trial management system.

Inclusion criteria were 50–74 years of age and postmenopausal status. Exclusion criteria were bilateral oophorectomy, previous ovarian or active non-ovarian malignancy, or increased familial ovarian cancer risk.

Ethical approval was provided by the UK North West Multicentre Research Ethics Committees (00/8/34) on June 23, 2000. All participants provided written informed consent. The trial design has been previously published and the protocol is available online.^9^, ^10^, ^11^, ^12^

### Randomisation and masking

The trial management system confirmed eligibility and then randomly allocated women using the Visual Basic randomisation statement and the RND function to annual screening using the MMS or USS strategy, or no screening in a 1:1:2 ratio. The trial management system allocated a set of 32 random numbers to each regional centre, of which eight were allocated to MMS, eight to USS, and the remaining 16 to no screening. We randomly allocated each successive volunteer within the regional centre to one of the numbers and subsequently randomly allocated them into a group. Investigators and participants were aware of group allocation but members of the outcomes committee were masked to randomisation group.

### Procedures

Annual screening in the MMS group used serum CA125 measurements, with the pattern over time interpreted using the risk of ovarian cancer (ROCA) calculation,^13^ which identifies significant rises in CA125 concentration above baseline. On the basis of risk, women were triaged to normal (annual screening), intermediate (repeat CA125 ROCA test in 3 months), and elevated (repeat CA125 ROCA test and transvaginal USS as a second-line test in 6 weeks) risk. Annual screening in the USS group used TVS as the primary test, which was classified as normal (annual screening), unsatisfactory (repeat in 3 months), or abnormal (scan with a senior ultrasonographer within 6 weeks). In both groups, women with persistent abnormalities were assessed by a trial clinician and referred to the NHS where they underwent further investigation or surgery. We deemed women who had surgery or a biopsy for suspected ovarian cancer after clinical assessment as screen positive.

Women were linked using their NHS number to national cancer and death registration data and Hospital Episode Statistics records (appendix p 4). An additional questionnaire was sent in June, 2020, to a subset of participants who had either exited the national registries or for whom it was not possible from Hospital Episode Statistics data to ascertain if both ovaries had been removed.

Throughout the trial, we interrogated the available sources to identify women diagnosed with any of 19 International Classification of Diseases (ICD)-10 codes for possible ovarian or tubal cancer and retrieved copies of medical notes.^12^, ^14^ The only exception was women with malignant neoplasm without specification of site (ICD-10 C80), who also had another non-ovarian, tubal, or peritoneal cancer registration. Medical notes, with any mention of randomisation group redacted, were reviewed by the outcomes review committee consisting of gynaecological pathologists and oncologists (NS, RM, RW, RA, LC, AS, and KW). The outcomes review committee assigned cancer site (ie, whether ovarian, tubal, or other site using a previously audited prespecified algorithm),^14^ Federation of Gynecology and Obstetrics (FIGO) 2014 stage, grade, morphology, ovarian cancer type, and cause of death. We defined ovarian and tubal cancer using the WHO 2014 classification^15^, ^16^ and death due to ovarian and tubal cancer based on disease progression (new or increases in size of previously documented lesions on imaging, clinical worsening, or rising biomarker concentrations). In the WHO 2014 classification, the definition for primary peritoneal cancers was revised. The outcomes review committee chair (NS) reviewed all 41 cancers previously classified as primary peritoneal as per WHO 2003 classification.^9^ The outcomes review committee re-staged all ovarian and tubal cancers using FIGO 2014 criteria (previously staged using FIGO 2003) diagnosed in 2001–14.

### Outcomes

The primary outcome was death due to ovarian (ICD-10 C56) or tubal (ICD-10 C57·0) cancer. Ovarian cancer includes primary non-epithelial ovarian cancer, borderline epithelial ovarian cancer, and invasive epithelial ovarian cancer. As stated above, ovarian cancer was defined using the revised WHO 2014 definition.^15^, ^16^ The site assignment is in contrast to the previous mortality analysis (censorship Dec 31, 2014), which used the WHO 2003 definition.^17^ The majority (40 of 41) of previously classified primary peritoneal cancers using WHO 2003 criteria were reclassified as ovarian or tubal cancers. Secondary outcomes were ovarian and tubal cancer incidence and stage. For all outcomes, subgroup analysis was undertaken for invasive epithelial ovarian and tubal cancer. All outcome data were kept confidential until unmasking. Case fatality rate by stage was a post-hoc outcome.

### Statistical analysis

At previous analysis (censorship Dec 31, 2014), there were 358 ovarian and tubal cancer deaths in the no screening group.^9^ Compared with the no screening group, the mean estimated relative mortality reduction in deaths was 11% (Cox model p=0·24) in the MMS group and 9% (Cox model p=0·32) in the USS group. Any mortality reduction was only apparent about 7 years after randomisation. 162 (45%) of 358 of the deaths in the no screening group during 2001–14 occurred before 7 years. In 2015, for the no screening versus MMS or USS comparisons, we estimated that an additional 233 no screening group events would give 80% power at a two-sided 5% significance level for a difference in relative mortality of 25% during long-term (2015–20) follow-up, conditional on the observed mortality reduction of 11%. This estimate translated to a target sample size of 591 overall events in the no screening group: all 233 new and 73% (431 of 591) of total no screening group events would occur beyond 7 years. No formal adjustment was made to the test for having previously analysed the data in 2015 or making two screen group comparisons. Instead, we decided to openly describe the multiplicity issues and acknowledge the unadjusted p values. As the number of events were less than anticipated on the planned censorship date of Dec 31, 2018, follow-up was extended with a new censorship date of June 30, 2020.

Descriptive statistics regarding ovarian cancer death and incidence were created including tabulations of histology, stage, and screen type by group. The primary mortality analysis was changed from the 2016 report, in which we used a Cox version of the log-rank test,^9^ which is most powerful under proportional hazards. For the current analysis, we extensively discussed the best approach within trial management and trial steering committees, and consulted 12 independent international statistical, trial, and screening experts. The details and rationale underpinning this important change are reported separately.^18^ In short, given the accumulating external evidence of delayed mortality effects in screening trials, most experts supported the change in primary analysis to a test that was sensitive to delayed effects. We chose the versatile test that was agnostic to the specific form of the screening effect. The versatile test, described in 2016,^18^ is a combination test of three log-rank test statistics (*Z*_1_, *Z*_2_, *Z*_3_), covering early, constant, and late effects respectively (appendix p 3).

All analyses were by intention to screen. The primary mortality analysis was an MMS versus no screening and USS versus no screening analysis of the primary outcome using the versatile test,^19^ with a Royston-Parmar model to estimate survival differences. We defined survival time from date of randomisation (*t*_0_=0) to date of death due to ovarian or tubal cancer or censorship, or sooner if the volunteer died of another cause or was lost to follow-up. No allowances were made for screening non-compliance (study groups) or contamination (no screening group). We describe potential time-dependent features of the screening effect by estimating the hazard ratio (HR) and the absolute survival difference at the prespecified timepoints of 5, 10, 15, and 18 years (maximal follow-up was 19·3 years) using a flexible parametric Royston-Parmar model (appendix p 3).

We undertook two secondary analyses of the primary outcome. We fitted a proportional hazards Cox model to the primary outcome data. To allow for a formal analysis of the late effects of ovarian cancer, not subject to issues of data re-use and multiple testing, we also fitted a Cox model to the new data acquired since Jan 1, 2015. Both the methods and results of sensitivity analyses are detailed in the appendix (pp 8–9). Survival from diagnosis in women with ovarian and tubal cancer in the no screening group was also compared to national age and period adjusted survival rates at 1, 5, and 10 years. We undertook a subgroup analysis using the versatile test of invasive epithelial ovarian and tubal cancer death, in which other ovarian cancers were censored at death.

For the secondary outcome, cumulative incidence of ovarian and tubal cancer were presented graphically using standard Kaplan-Meier methods, based on time from randomisation to diagnosis. Death from other causes and bilateral salpingo-oophorectomy were censoring events. Administrative censorship was the same as for the mortality analysis (June 30, 2020). Ovarian and tubal cancer incidence rates were explored parametrically using a Royston-Parmar model that specifically allowed exploration of the underlying hazard functions for cancer incidence. For the secondary outcome of ovarian and tubal cancer incidence by stage, and the subgroup analysis of invasive epithelial ovarian and tubal cancers, we used incidence rate ratios with 95% CIs to compare no screening versus MMS and USS groups separately. We also calculated stage-specific ovarian and tubal cancer case fatality rates.

We used Stata, version 16 (versatile test verswlr function) for all statistical analyses. Results were independently verified by a different statistician using R, version 4.0.2 (versatile test logrank.maxtest of nph package). This trial is registered with ISRCTN, 22488978, and ClinicalTrials.gov, NCT00058032.

### Role of the funding source

The funders of the study had no role in study design, data collection, data analysis, data interpretation, or writing of the report.

## Results

**Figure 1:**
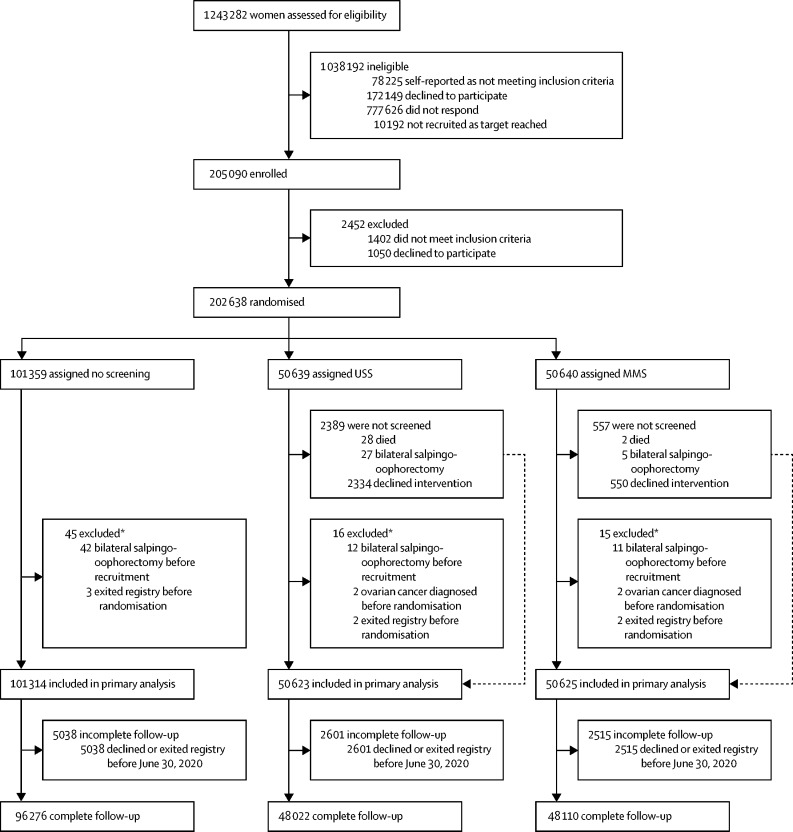
Trial profile MMS=multimodal screening. USS=ultrasound screening. *Events occurred before recruitment, but were discovered after randomisation.

Between April 17, 2001, and Sept 29, 2005, we invited 1 243 282 women to participate and randomly assigned 202 638 (16·3% of 1 243 282; figure 1).^10^ We followed-up participants until June 30, 2020. The final cohort eligible for analysis consisted of 202 562 (>99·9%) of 202 638 women: 50 625 (>99·9%) in the MMS group, 50 623 (>99·9%) in the USS group, and 101 314 (>99·9%) in the no screening group. We excluded 76 (<0·1%) women (15 [<0·1%] in the MMS group; 16 [<0·1%] in the USS group; and 45 [<0·1%] in the no screening group; figure 1) as they had bilateral salpingo-oophorectomy, ovarian cancer before joining the trial, or had exited the registry before randomisation. As previously reported, baseline characteristics were balanced between the groups.^9^ Screening ended on Dec 31, 2011. We undertook 673 345 annual screens: 345 570 in the MMS group and 327 775 in the USS group. Compliance with screening was high (81% in the MMS group and 78% in the USS group) with women undergoing a median of eight annual screens.^9^

After the end of annual screening on Dec 31, 2011, all women were followed up until the censorship date of June 30, 2020. Complete follow-up until censorship or death was possible in 192 478 (95·0%) women (48 110 [95·0%] in the MMS group; 48 022 [94·9%] in the USS group; and 96 276 [95·0%] in the no screening group) resulting in 3·16 million women-years. Median follow-up was 16·3 years (IQR 15·1–17·3) for all groups.

**Figure 2:**
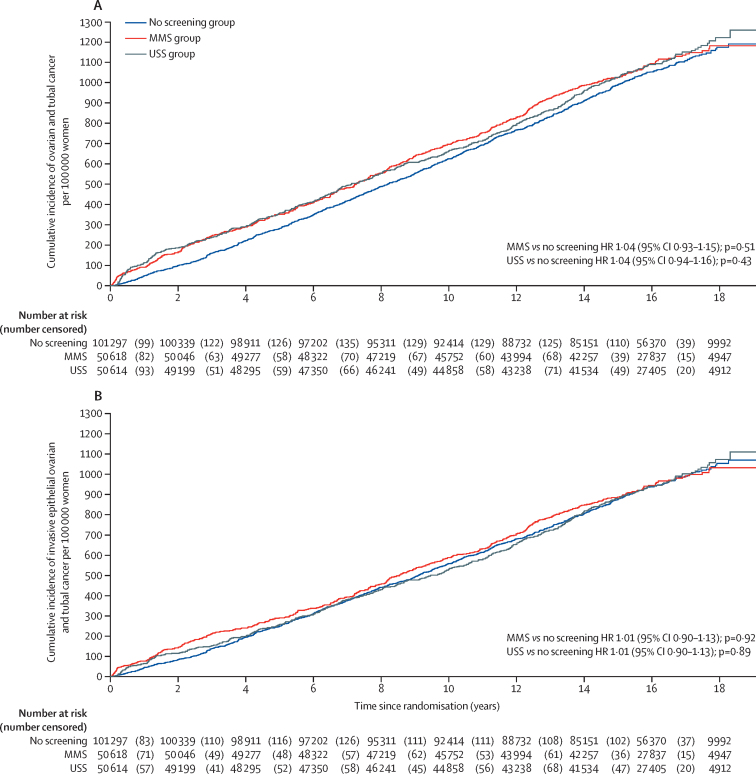
Kaplan-Meier cumulative incidence per 100 000 women for all ovarian and tubal cancers (A) and for invasive epithelial ovarian and tubal cancers (B) MMS=multimodal screening. USS=ultrasound screening. HR=hazard ratio.

We identified 4482 women with the 19 prespecified ICD-10 codes for possible ovarian and tubal cancer who were reviewed by the outcomes review committee (appendix p 5). Of them, 2055 (45·9%) were confirmed to have ovarian or tubal cancer (table 1). The incidence of ovarian and tubal cancer per 100 000 women-years was 67·7 (95% CI 61·9–73·5; 522 cancers; 770 967 women-years) in the MMS group, 68·2 (62·4–74·1; 517 cancers; 755 677 women-years) in the USS group, and 65·4 (61·4–69·4; 1016 cancers; 1 552 703 women-years) in the no screening group (appendix p 11). Figure 2A provides Kaplan-Meier cumulative cancer rates for all women and figure 2B provides Kaplan-Meier cumulative cancer rates for invasive epithelial ovarian and tubal cancers. Both plots show a greater number of cancer diagnoses in the screening groups during the first screening year, reflecting the lead time to diagnosis achieved by screening. The difference was largely maintained throughout the screening phase before apparent catch-up by the no screening group during the extended period of follow-up after the end of screening. However, the pattern of catch-up in the USS group was less pronounced, and this observation is elucidated by the Royston-Parmar model hazard functions (appendix pp 19–20), in which the rate of cancer incidence drops below the no screening group between years 4–14 approximately, before rising back above the no screening group rate.

**Table 1:** Ovarian and tubal cancers grouped by primary site and screening status

		**Total**	**Screen positives**	**Cancers not detected by screening**			
				Screen negatives ≤1 year from last test of screening episode	Screen negatives >1 year after last test of screening episode	Never attended screening	Diagnosed >1 year after end of screening*
**Multimodal screening (50 625 women, 789 129 women-years)**							
Ovarian and tubal cancer		522 (100%)	212 (41%)	41 (8%)	41 (8%)	3 (1%)	225 (43%)
	Non-epithelial ovarian cancer	16 (100%)	7 (44%)	2 (13%)	2 (13%)	0	5 (31%)
	Borderline epithelial ovarian cancer	54 (100%)	24 (44%)	10 (19%)	5 (9%)	0	15 (28%)
	Invasive epithelial ovarian and tubal cancer	452 (100%)	181 (40%)	29 (6%)	34 (8%)	3 (1%)	205 (45%)
**Ultrasound screening (50 623 women, 790 231 women-years)**							
Ovarian and tubal cancer		517 (100%)	164 (32%)	63 (12%)	50 (10%)	19 (4%)	221 (43%)
	Non-epithelial ovarian cancer	13 (100%)	11 (85%)	0	1 (8%)	0	1 (8%)
	Borderline epithelial ovarian cancer	59 (100%)	48 (81%)	2 (3%)	1 (2%)	3 (5%)	5 (8%)
	Invasive epithelial ovarian and tubal cancer	445 (100%)	105 (24%)	61 (14%)	48 (11%)	16 (4%)	215 (48%)
**No screening (101 314 women, 1 577 517 women-years)**							
Ovarian and tubal cancer		1016† (100%)	..	..	514 (51%)	..	499 (49%)
	Non-epithelial ovarian cancer	17 (100%)	..	..	7 (41%)	..	10 (59%)
	Borderline epithelial ovarian cancer	91 (100%)	..	..	50 (55%)	..	41 (45%)
	Invasive epithelial ovarian and tubal cancer	905 (100%)	..	..	457 (50%)	..	448 (50%)

Data are n (%).

* Screening end Dec 31, 2011.

† Includes one case in which histology was not available and two cases of neoplasm of uncertain or unknown behaviour.

Overall, 1805 (87·8%) of 2055 women (452 [0·9%] in the MMS group; 445 [0·9%] in the USS group; 905 [0·9%] in the no screening group) had invasive epithelial ovarian or tubal cancers. The proportion of the most aggressive type II cancers (79·2% in the MMS group; 82·2% in the USS group; and 76·4% in the no screening group) was similar across the groups (appendix p 6). At 9·5 years after the end of screening, compared with the no screening group, there was a 47·2% (95% CI 19·7 to 81·1) higher incidence of stage I disease and a 24·5% (−41·8 to –2·0) lower incidence of stage IV disease in the MMS group (table 2). Overall, there was a 39·2% (95% CI 16·1 to 66·9) higher incidence of stage I or II disease and 10·2% (−21·3 to 2·4) lower incidence of stage III or IV disease in the MMS group compared with the no screening group. For the subgroup analysis of invasive epithelial ovarian and tubal cancers, the changes in stage distribution in the MMS group compared with the no screening group persisted. There was no evidence of a change in incidence in any stage in the USS group compared with the no screening group.

**Table 2:** Summary of incidence and case fatality rate by FIGO 2014 stage for ovarian and tubal cancer

		**FIGO 2014 stage**					**Total**
		I	II	III	IV	Unable to stage	
Ovarian and tubal cancers (WHO 2014 classification)*							
No screening							
	Cases	212 (20·9%)	73 (7·2%)	510 (50·2%)	208 (20·5%)	13 (1·3%)	1016
	Deaths	20 (9·4%)	24 (32·9%)	391 (76·7%)	174 (83·7%)	10 (76·9%)	609 (59·9%)
MMS							
	Cases	155 (29·7%)	42 (8·0%)	242 (46·4%)	78 (14·9%)	5 (1·0%)	522
	Deaths	23 (14·8%)	16 (38·1%)	190 (78·5%)	62 (79·5%)	4 (80·0%)	291 (55·7%)
USS							
	Cases	121 (23·4%)	36 (7·0%)	253 (48·9%)	105 (20·3%)	2 (0·4%)	517
	Deaths	8 (6·6%)	6 (16·7%)	188 (74·3%)	88 (83·8%)	2 (100·0%)	290 (56·1%)
Between group differences in cases compared with no screening at 9·5 years after end of screening†							
	MMS	47·2% (19·7 to 81·1)	15·9% (−20·7 to 69·4)	−4·4% (−18·0 to 11·4)	−24·5% (−41·8 to −2·0)	..	..
	USS	17·0% (−6·4 to 46·2)	1·1% (−32·2 to 50·6)	1·7% (−12·6 to 18·2)	3·4% (−18·2 to 30·8)	..	..
Invasive epithelial ovarian and tubal cancers (WHO 2014 classification)*							
No screening							
	Cases	116 (12·8%)	69 (7·6%)	501 (55·3%)	208 (23·0%)	12 (1·3%)	906
	Deaths	18 (15·5%)	24 (34·8%)	391 (78·0%)	174 (83·7%)	10 (83·3%)	617 (68·1%)
MMS							
	Cases	91 (20·1%)	41 (9·1%)	237 (52·4%)	78 (17·3%)	5 (1·1%)	452
	Deaths	22 (24·2%)	16 (39·0%)	190 (80·2%)	62 (79·5%)	4 (80·0%)	294 (65·0%)
USS							
	Cases	55 (12·4%)	35 (7·9%)	249 (56·0%)	104 (23·4%)	2 (0·4%)	445
	Deaths	7 (12·7%)	6 (17·1%)	186 (74·7%)	87 (83·7%)	2 (100·0%)	288 (64·7%)
Between group differences in cases compared with no screening at 9·5 years after end of screening†							
	MMS	52·2% (16·8 to 98·4)	15·8% (−19·4 to 66·4)	−4·8% (−18·3 to 10·9)	−23·7% (−40·7 to −1·7)	..	..
	USS	−8·0% (−32·7 to 25·7)	−5·2% (−35·8 to 39·9)	0·5% (−13·6 to 16·8)	−0·8% (−21·3 to 25·1)	..	..

Data for cases are n (%); data for deaths are n (case fatality rate for stage); data for between group differences in cases are percentage (95% CI). FIGO=Federation of Gynecology and Obstetrics. MMS=multimodal screening. USS=ultrasound screening.

* Includes cases previously designated as primary peritoneal cancer as per WHO 2003 classification.

† Between group differences from a poisson model with length of analysis time as exposure variable; percentage difference taken from the incidence rate ratio, where percentage difference equals incidence rate ratio minus 1 multiplied by 100%.

**Figure 3:**
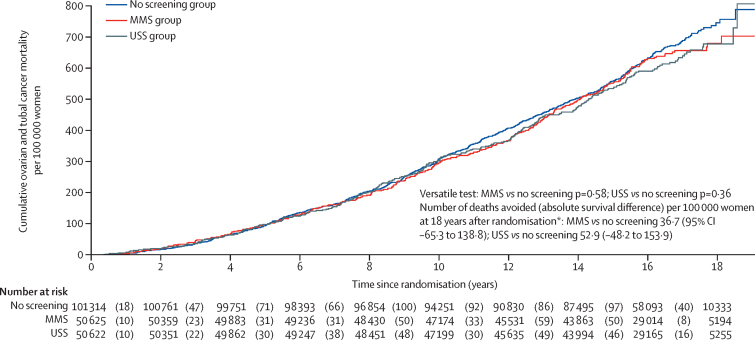
Kaplan-Meier cumulative mortality for ovarian and tubal cancer per 100 000 women MMS=multimodal screening. USS=ultrasound screening. *Royston-Parmar model based estimates of the effect of screening (appendix p 10).

At censorship, 1206 (0·6%) women had died of ovarian cancer: 296 (0·6%) of 50 625 in the MMS group, 291 (0·6%) of 50 623 in the USS group, and 619 (0·6%) of 101 314 in the no screening group. Ovarian and tubal cancer deaths and incidence by year from randomisation is detailed in the appendix (p 7). The versatile test (primary analysis) showed that there was no evidence of a reduction in ovarian and tubal cancer deaths in either the MMS (p=0·58) or USS (p=0·36) group compared with the no screening group (table 3). Figure 3 shows the Kaplan-Meier cumulative death rates, with any divergence between the screen and no screen groups being minimal. A sensitivity analysis that only considered data obtained by equivalent means of electronic health records across all three groups also showed no evidence of a difference using the versatile test result for both the MMS (p=0·60) and USS (p=0·37) group (appendix p 9). This analysis and other sensitivity analyses are detailed in the appendix (pp 8–9). A Cox model (secondary analysis) estimated a HR of 0·96 (95%CI 0·83–1·10) for MMS versus no screening and 0·94 (0·82–1·08) for USS versus no screening. A Cox model fitted only to data from 2015 onwards (secondary analysis) estimated a HR of 1·05 (95%CI 0·86–1·30) for MMS versus no screening and 0·99 (0·80–1·22) for USS versus no screening.

**Table 3:** Summary of analyses of relative reduction of ovarian and tubal cancer deaths

	**Number of women (n)**	**Deaths (n)**	**Maximum χ^2^ or mortality reduction (hazard ratio)**	**p value**
Primary analysis*(ovarian and tubal cancer deaths)				
MMS	50 625	296	0·41	0·58
USS	50 622	291	1·23	0·36
No screening	101 314	619	..	..
Subgroup outcome analysis*(invasive epithelial ovarian and tubal cancer)				
MMS	50 625	295	0·41	0·60
USS	50 622	287	1·37	0·33
No screening	101 314	617	..	..
**Secondary analysis**†**(all data 2001–20)**				
MMS	50 625	296	0·96 (0·83 to 1·10; 0·068)	0·52
USS	50 622	291	0·94 (0·82 to 1·08; 0·067)	0·37
No screening	101 314	619	..	..
**Secondary analysis**†**(only data 2015–20)**				
MMS	45 999	136	1·05 (0·86 to 1·30; 0·112)	0·63
USS	46 079	128	0·99 (0·80 to 1·22; 0·107)	0·91
No screening	91 808	258	..	..

Data are maximum χ^2^ for primary and subgroup analyses or mortality reduction (95% CI; SE) for secondary analyses. MMS=multimodal screening. USS=ultrasound screening.

* Versatile test.

† Cox model.

For the secondary outcome of invasive epithelial ovarian and tubal cancers, there were 295 (0·6%) deaths in the MMS group, 287 (0·6%) in the USS group, and 619 (0·6%) in the no screening group (table 3). The cumulative death rates similarly showed no evidence of an effect of screening (appendix p 11). The versatile test for mortality reduction showed no evidence of difference in both the MMS group (p=0·60) and the USS group (p=0·33). The appendix shows the Royston-Parmar model fit to the non-parametric Kaplan-Meier curves (pp 12–13) and the associated hazard functions for each group (pp 14–15). All hazard functions were consistently increasing with only small differences between the screening and no screening groups. At 18 years after randomisation, the Royston-Parmar model estimates of survival differences per 100 000 women were 36·7 (95% CI –65·3 to 138·8) for MMS compared with no screening and 52·9 (−48·2 to 153·9) for USS compared with no screening (figure 3, appendix p 10).

Compared with no screening, in the MMS group, we observed a higher ovarian and tubal cancer case fatality rate in patients with stage I disease (20 [9·4%] of 212 in the no screening group *vs* 23 [14·8%] of 155 in the MMS group) and a lower rate with stage IV disease (174 [83·7%] of 208 in the no screening group *vs* 62 [79·5%] of 78 in the MMS group), which persisted on subgroup analysis of invasive epithelial ovarian cancer. In the USS group, the stage-specific case fatality rates appear to be similar to the no screening group, except for stage II, but numbers in this group are small (table 2).

Survival from diagnosis in women with ovarian and tubal cancer in the no screening group was better in comparison to national age and period adjusted survival rates (1 year 77% *vs* 68%; 5 year 40% *vs* 37%; appendix p 18).

## Discussion

Our results from the largest ovarian cancer screening trial to date show that on long-term follow-up (median 16·3 years after randomisation), neither MMS or USS, as used in UKCTOCS, significantly reduced deaths from ovarian and tubal cancer. There was a 47·2% higher incidence of stage I cancer and 24·5% lower incidence of stage IV cancer, resulting in an overall 39·2% higher incidence of stage I or II cancer and 10·2% lower incidence of stage III or IV cancer in the MMS group than in the no screening group. General population screening for ovarian and tubal cancer with either of the screening strategies cannot be recommended based on evidence to date. The changes in stage distribution in the MMS group did not translate into mortality reduction, emphasising the importance of having disease-specific mortality as the primary outcome in ovarian cancer screening trials.

All women were treated within the NHS where, since 2004, patients are managed within a designated cancer-specific multidisciplinary team setting and ovarian cancer surgery is done only in gynaecological oncology cancer centres by subspecialty trained and accredited gynaecological oncologists. Therefore, there is unlikely to be variability in quality of care between the randomisation groups. Detailed analysis of stage and histology specific treatment is underway and will be the subject of a future report.

Achieving a mortality reduction will require a screening strategy that can detect ovarian and tubal cancer even earlier and in a larger proportion of women than we were able to achieve. Our findings make it even more important that before general population screening is introduced, any new test is shown to reduce ovarian and tubal cancer deaths in a future randomised controlled trial. These trials take many years to complete but the high compliance with annual screening in UKCTOCS suggests that women are very motivated to join them. Given that such trials take considerable time, it is probable that population screening for ovarian cancer is more than a decade away.

It is difficult to extrapolate these results to ovarian cancer screening of women at high-risk, in which the strategy has involved MMS once every 3–4 months alongside risk-reducing surgery and resulted in a significant reduction in the proportion of women diagnosed with advanced disease.^20^, ^21^ Biological differences also exist between cancers in women with *BRCA* gene mutations and the general population, which result in improved treatment responses in carriers of *BRCA* mutations. Unfortunately, it is unlikely that the true effect of screening on mortality will ever be assessed in this population as a randomised controlled trial is challenging, and potentially very effective preventive measures such as risk reducing salpingectomy with delayed oophorectomy are being evaluated.

Our results have implications for ovarian cancer symptom awareness campaigns as most women who were screen-detected had no high-alert symptoms^22^ and were diagnosed earlier than would have been possible with a symptom-based approach. Our findings suggest that earlier diagnosis of invasive epithelial ovarian and tubal cancer in the symptomatic population is unlikely to translate into reduced mortality. However, it is important to note that there have been substantial advances in the treatment of advanced disease in the past 10 years since the end of screening in Dec 31, 2011. The advances in treatment, in combination with earlier diagnosis could contribute to better quality of life and improved outcomes. In addition, achieving a rapid diagnosis is of great importance to women and their families.

Our mortality results are similar to those reported for ovarian cancer screening in the Prostate Lung Colorectal Ovarian (PLCO) cancer screening trial, the only other large randomised controlled trial to report on the effects of ovarian cancer screening on mortality.^7^, ^8^ In the PLCO trial, no evidence was seen of a reduction in ovarian cancer deaths between the screen and no screen arms, either at median follow-up of 12·4^7^ or 14·7^8^ years. However, in UKCTOCS, we found a higher incidence of stage I and lower incidence of stage IV disease in the MMS group than in the no screening group. The PLCO trial found no evidence of a difference in stage distribution between the screened and non-screened groups.^7^, ^8^ The use of a longitudinal CA125 algorithm in the MMS group instead of a single CA125 cutoff, as in the PLCO trial, might have contributed to this difference. We have previously shown that a longitudinal algorithm allows us to detect disease earlier and with greater sensitivity than a single CA125 cutoff.^23^, ^24^

Despite the 24·5% reduction in stage IV disease in the MMS group, the overall reduction in stage III or IV incidence 9·5 years after end of screening was only 10%, with little change in stage III incidence. Previous reports have highlighted the need for a large reduction in late-stage incidence as a prerequisite for reducing cancer mortality. However, it should be noted that the length of follow-up and, therefore, the dilution effect in the screen groups of inclusion of cancers diagnosed clinically after end of screening varied in these reports. An analysis of breast cancer screening trials found no reduction in breast cancer mortality in trials that achieved less than 10% reduction in stage III or IV disease and a mean reduction of 28% in trials that saw a 20% or greater reduction.^25^ In colorectal and lung cancer screening, much of the mortality reduction is related to reductions in stage IV disease, which has much higher mortality than earlier stages.^26^, ^27^ For ovarian and tubal cancer, the high mortality associated with stage III and IV disease, combined with most women clinically detected with stage III disease, requires a substantial reduction in the incidence of both stages before a mortality reduction is possible.

There are previous instances in which increased incidence of stage I or II cancers in the screen group in screening trials did not translate to a mortality reduction. In the four early randomised controlled trials of lung cancer,^28^ compared with the control group, the screened groups showed significant improvements in stage distribution. However, much of the screen-detected early stage disease cases in these trials were indolent. This finding in the lung cancer trials was accompanied by a significant increase in cancer incidence in the screen groups, but no reduction in late-stage incidence. Both together suggest that overdiagnosis was the main contributor to the absence of reduction in mortality.^28^ This result differs from UKCTOCS, in which no significant increase in cancer incidence was observed in either of the screen groups. In the MMS group of UKCTOCS, it seems probable that the cancers shifted to an earlier stage had an intrinsic poor prognosis, which was not altered by earlier detection and the available treatments for early stage disease. Further histopathological and genetic analysis of these early stage screen-detected cancers could yield important information about the biology of ovarian cancer.

The 47·2% increase in incidence of stage I and 24·5% decrease in incidence in stage IV disease was accompanied by a higher stage I and lower stage IV case fatality rate in the MMS group. This finding persisted in subgroup analysis of invasive epithelial cancers. The findings are unlike that described previously in cancer screening trials.^26^ The result suggests that in the MMS group, although earlier detection of stage IV invasive epithelial ovarian and tubal cancers improved outcomes, earlier detection in stage I cancers that might have presented in later stages in the absence of screening, did not have the same effect. Stage-specific mortality and treatment will be the subject of further in-depth analyses, which will be reported in a separate publication.

The key strengths of UKCTOCS have been previously detailed^9^ and include scale; multicentre design; adherence to protocol through use of a bespoke web-based trial management system with automation of key processes, remote data entry, and concurrent central monitoring; completeness of follow-up through linkage to national registries and administrative databases; and independent assignment of site and cause of death. The longitudinal algorithm we used since 2001 to interpret CA125 concentrations was innovative and forward thinking. The UK Government's Accelerating Detection of Disease Programmme includes collection of repeat biological samples that will enable building of such algorithms. We re-staged all cases using the latest FIGO 2014 criteria and revised our ovarian and tubal cancer site assignment using WHO 2014 classification to reflect current understanding of disease biology. We also changed our primary analysis approach from a constant-effect approach (proportional hazards Cox-model) to one that allows for a delayed effect (the versatile test) to reflect growing evidence that the mortality reduction in cancer-screening trials, if present, is delayed. We did this through a transparent process with publication of our methods and the expert opinions that underpinned our decision.^18^

Much has been learnt from the design, conduct, and analysis of UKCTOCS, which is relevant to future large-scale trials. In addition, a large bioresource of serum samples (>550 000) and linked data (UKCTOCS Longitudinal Women's Cohort) has been built through the generosity of the participants. The resource includes a rare sample set of up to 11 annual blood samples from women in the MMS group. In ovarian cancer, it has allowed us to collaborate to explore new biomarkers and develop longitudinal algorithms.^24^ The data provides a unique opportunity to study the natural history of ovarian and tubal cancer. Important research is also underway on early detection of other cancers and risk stratification in cardiovascular disease using this bioresource.

A key limitation of the trial is the interval from end of screening in 2011 to censorship in 2020, which raises the possibility of dilution of the screening effect. However, extended follow-up after screening is the norm in screening trials and did not affect mortality reduction seen in the European Randomized Study of Screening for Prostate Cancer.^29^ Also, there was no variation in the ovarian cancer mortality HR at 15 years and 18 years from randomisation in our Royston-Parmar model (appendix p 10).

In addition, most screen-detected women were diagnosed and treated more than a decade ago, before many of the advances in clinical management (eg, widespread use of ultraradical surgery, earlier treatment modulation based on better prognostic indicators, and targeted therapies), which could have improved outcomes. In retrospect, second-line tests could have been further optimised so that time to diagnosis after an abnormal screen was reduced.^23^ Finally, clinicians could have been encouraged to intervene earlier when faced with rising biomarker concentrations and normal imaging.^30^ This theory is supported by modelling, which suggests that the majority of stage I, type II epithelial cancers are 0·4–1·3 cm in diameter and, therefore, difficult to reliably image.^31^

We began our mission to reduce deaths from ovarian cancer in the 1980s,^5^, ^6^ started our pilot randomised controlled trial in the mid-1990s,^32^ and then undertook UKCTOCS over the next 20 years. The journey has involved more than 200 000 women who have trusted us and generously given their time, multiple UK funding agencies, numerous NHS staff both at the trial centres and more widely, and the support of many UK and international charities, and expert groups. To them, we are hugely grateful. While disappointing, the trial has provided a clear answer that our screening strategies coupled with treatment protocols available in 2001–11 (the active screening phase) did not save lives. Currently, general population screening for ovarian and tubal cancer cannot be recommended. We remain optimistic that further research will develop more effective ways to detect and treat this lethal disease. Meanwhile, the UKCTOCS biorepository with longitudinal samples provides a unique opportunity to advance early detection biomarker research.

## Data sharing

The protocol is available on the study website. The individual participant data that underlie the results reported in this Article, after de-identification, will be available beginning 12 months after publication. Researchers will need to state the aims of any analyses and provide a methodologically sound proposal. Proposals should be directed to u.menon@ucl.ac.uk. Data requestors will need to sign a data access agreement and in keeping with patient consent for secondary use, obtain ethical approval for any new analyses.

## Declaration of interests

UM has stock ownership awarded by University College London (UCL) in Abcodia, which holds the licence for ROCA. She has received grants from the Medical Research Council (MRC), Cancer Research UK, the National Institute for Health Research (NIHR), and The Eve Appeal. She holds patent number EP10178345.4 for Breast Cancer Diagnostics. MP has received grants and AG-M, MB, AR, CK, GC, and SKM have been funded by grants from MRC, Cancer Research UK, NIHR, and The Eve Appeal. RM has received grants from The Eve Appeal, Rosetrees Charity, and Barts Charity, and personal fees from AstraZeneca. SJS holds the (expired) patent for ROCA, patented and owned by Massachusetts General Hospital and Queen Mary University of London, licenced to Abcodia. He reports stock options from SISCAPA Assay Technologies, and personal fees from Abcodia, Guardant Health, and Freenome, outside the submitted work. IJJ reports grants from Eve Appeal Charity, Medical Research Council, Cancer Research UK, and NIHR during the conduct of the study. He co-invented the ROCA in 1995, it was patented by Massachusetts General Hospital and Queen Mary University of London and is owned by these universities. Massachusetts General Hospital and Queen Mary University of London granted a licence to ROCA to Abcodia in 2014. IJJ is non-executive director, shareholder, and consultant to Abcodia and has rights to royalties from sales of the ROCA. He founded (1985), was a trustee of (2012–14), and is now an Emeritus trustee (2015–present) of The Eve Appeal, one of the funding agencies for UKCTOCS. All other authors declare no competing interests.
