# Supplementary material for: Ovarian cancer population screening and mortality after long-term follow-up in the UK Collaborative Trial of Ovarian Cancer Screening (UKCTOCS): a randomised controlled trial
Source: Lancet. 2021 Jun 5;397(10290):2182–93. doi: 10.1016/S0140-6736(21)00731-5 (PMC8192829; doi:10.1016/S0140-6736(21)00731-5)
Supplement: Supplementary appendix [file mmc1.pdf]

THE LANCET

Supplementary appendix

This appendix formed part of the original submission and has been peer reviewed. We post it as supplied by the authors.

Supplement to: Menon U, Gentry-Maharaj A, Burnell M, et al. Ovarian cancer population screening and mortality after long-term follow-up in the UK Collaborative Trial of Ovarian Cancer Screening (UKCTOCS): a randomised controlled trial. *Lancet* 2021; published online May 12. http://dx.doi.org/10.1016/S0140-6736(21)00731-5.

Appendix: Supplementary Material

Contents

A. Further details of statistical analysis 3

B. Supplementary Tables..4

Web Table 1: Source of notification of cancer and deaths of women whose notes were submitted for Outcome Review...4

Web Table 2: Diagnosis assigned by Outcome Review Committee for women with pre-specified ICD-10 codes...5

Web Table 3: Detailed morphology and FIGO 2014 stage of ovarian and tubal cancers ...6

Web Table 4: Ovarian and tubal cancer incidence and deaths by group and year of analysis...7

Web Table 5: Sensitivity analysis for primary analysis 8

Web Table 6: Royston-Parmar model based estimates of the effect of screening at the pre-specified time-points of 5, 10, 15 and 18 years since randomisation 10

C. Supplementary Figures 11

Web Figure 1: Cumulative invasive epithelial ovarian and tubal cancer deaths 11

Web Figure 2a: Cumulative ovarian and tubal cancer deaths by randomisation group with RP model fit overlaid - MMS versus no screening 12

Web Figure 2b: Cumulative ovarian and tubal cancer deaths by randomisation group with RP model fit overlaid - USS versus no screening 13

Web Figure 3a: RP model estimate of ovarian and tubal cancer death rates (hazard function) with 95% confidence limits – MMS versus no screening....................................14

Web Figure 3b: RP model estimate of ovarian and tubal cancer death rates (hazard function) with 95% confidence limits – USS versus no screening.....................................15

Web Figure 4a: RP model estimate of ovarian and tubal cancer death hazard ratio for MMS versus no screening with 95% confidence limits 16

Web Figure 4b: RP model estimate of ovarian and tubal cancer death hazard ratio for USS versus no screening with 95% confidence limits 17

Web Figure 5: Survival rates in women with ovarian and tubal cancer in the no screening group compared with one-, and five- year age and period adjusted survival rates in the UK population. 10 year survival rates by age group not available for the UK population........18

Web Figure 6a: Cumulative incidence rates (hazard functions) of ovarian and tubal cancer with 95% confidence bands MMS versus no screening..19

Web Figure 6b: Cumulative incidence rates (hazard functions) of ovarian and tubal cancer with 95% confidence bands USS versus no screening...20

D. Long term follow up of UKCTOCS committees and teams...21

A. Further details of statistical analysis

We chose the Versatile test described in 2016[1] which is a combination test of 3 log-rank test statistics (Z_1, Z_2, Z_3), each weighted differentially. The first is unweighted and so is the standard log-rank test, whilst the latter two are separately weighted according to either the overall survival or failure function, and hence sensitive to early and late effects, respectively. The test does not anticipate a particular functional form for the hazard ratio, but allows for any non-proportional treatment effect (whether early or late) to be more likely identified than a standard log-rank test would, whilst still maintaining good power under proportional hazards. The test statistic $Z_m = \max\{|Z_1|, |Z_2|, |Z_3|\}$ was compared against a trivariate normal distribution with mean vector zero and a variance-covariance matrix based on the covariance estimates between the three test statistics. It is this correlation between tests that limits the effective degrees of freedom required to (considerably) less than 3, providing statistical efficiency whilst 'covering the bases' of possible outcomes.

We describe time-dependent features of the screening effect by estimating the hazard ratio and the absolute survival difference at the pre-specified time-points of *t*=5, 10, 15 and 18 years (maximal follow-up was 19·3 years) using a flexible parametric RP model. The number of knots and subsequent spline functions were chosen based on the model with the lowest Akaike Information Criteria (AIC) when fitted to the MMS group, and then applied to the other groups. Knot placement was the default option, according to the centiles of the uncensored MMS group survival times.

The model was fitted using the Stata package `stpm2`.[2]

References

1. Karrison TG. Versatile tests for comparing survival curves based on weighted log-rank statistics. *The Stata Journal* 2016; 16(3): 678-90.
2. Lambert PC RP. Further development of flexible parametric models for survival analysis. *The Stata Journal* 2009; 9(2): 265-90.

B. Supplementary Tables

Web Table 1: Source of notification of cancer and deaths of women whose notes were submitted for Outcome Review

Sources of cancer and death notification	Date last update received
Death Registration	
England	18/09/2020
Wales	18/09/2020
Northern Ireland	14/09/2020
Cancer Registration	
England	07/10/2020
Wales*	31/12/2016
Northern Ireland	23/03/2020
Hospital Episode Statistics	
England	05/06/2020
Wales	14/07/2020
Registry Flagging (Members & Posting)	
England	18/09/2020
Wales	18/09/2020
National Disease Registration Service	
England	13/02/2015
UKCTOCS Health Questionnaires	
FUQ1	2005-2010
FUQ2	04/04/2014
FUQ3**	16/06/2020
Ad-hoc Communication	
Trial participants, their families and physicians	Throughout the trial

FUQ=Follow-up questionnaire. *NHS Digital was not able to distribute Welsh cancer registration data following implementation of GDPR. **Sent to subset of participant who had exited registry flagging or required confirmation of bilateral oopherectomy

Web Table 2: Diagnosis assigned by Outcome Review Committee for women with pre-specified ICD-10 codes

Diagnosis assigned by Outcome Review Committee	All arms	No screening	MMS	USS
Total*	4482	2183	1170	1129
Ovarian or tubal cancer	2055	1016	522	517
Malignant neoplasm without specification of site	252	124	69	59
Malignant neoplasm without specification of site but not ovarian or tubal cancer	388	197	95	96
Other primary cancer	1591	766	439	386
Not a cancer	196	80	45	71

MMS=multimodal screening. USS=ultrasound screening. *women for whom notification was received of 19 pre-specified ICD-10 codes

Web Table 3: Detailed morphology and FIGO 2014 stage of ovarian and tubal cancers

Characteristics	MMS	USS	No screening	Total
**Morphology**				
**Ovarian and tubal cancers**	**522**	**517**	**1016***	**2055**
**Invasive epithelial ovarian and tubal cancers**	**452**	**445**	**905**	**1802**
Type I invasive epithelial cancer	64	45	115	224
Low grade serous	16	12	21	49
Low grade carcinoma	0	0	3	3
Mucinous	8	11	33	52
Low grade endometrioid	20	8	31	59
Clear Cell	19	14	25	58
Brenner	0	0	1	1
Mixed Cell	0	0	1	1
Small Cell	1	0	0	1
Type II invasive epithelial cancer	358	366	692	1416
High grade serous	300	309	586	1195
High grade carcinoma	27	32	50	109
High grade endometrioid	19	9	17	45
Carcinosarcoma	12	16	39	67
Type uncertain (grade unknown)	30	34	98	162
Serous	8	7	17	32
Carcinoma	22	27	79	128
Endometrioid	0	0	2	2
**Borderline epithelial ovarian cancer**	**54**	**59**	**91**	**204**
Serous	32	42	43	117
Mucinous	17	12	41	70
Endometrioid	2	1	1	4
Brenner	0	0	4	4
Mixed Cell	3	4	2	9
**Non-epithelial ovarian cancer**	**538**	**530**	**17**	**1085**
Sarcoma	1	2	0	3
Sex cord-stromal	13	8	14	35
Teratoma related	2	3	3	8
**Stage**				
**Invasive epithelial ovarian and tubal cancer**	**452**	**445**	**905**	**1802**
I	91	55	116	262
Ia	35	20	43	98
Ib	4	1	2	7
Ic	52	34	71	157
II	41	35	69	145
IIa	18	22	27	67
IIb	23	13	42	78
III	237	249	501	987
IIIa	16	16	25	57
IIIb	45	33	55	133
IIIc	176	200	421	797
IV	78	104	208	390
IVa	22	33	75	130
IVb	56	71	133	260
Unable to stage	5	2	11	18
**Borderline epithelial ovarian cancer**	**54**	**59**	**91**	**204**
I	50	55	79	184
II	0	1	3	4
III	4	3	9	16
**Non-epithelial ovarian cancer**	**16**	**13**	**17**	**46**
I	14	11	16	41
II	1	0	1	2
III	1	1	0	2
IV	0	1	0	1

Data are numbers. MMS=multimodal screening. USS=ultrasound screening. *Includes 1 case where histology not available and 2 cases of neoplasm of uncertain or unknown behaviour

Web Table 4: Ovarian and tubal cancer incidence and deaths by group and year of analysis

Time from randomisation	Ovarian and tubal cancer deaths				Ovarian and tubal cancer cases			
	no screening	MMS	USS	overall	no screening	MMS	USS	overall
0 ≤years <1	4	2	4	10	47	46	56	149
1 ≤ years <2	14	8	6	28	52	36	37	125
2 ≤ years <3	19	13	8	40	62	41	25	128
3 ≤ years <4	28	10	14	52	60	22	26	108
4 ≤ years <5	28	16	17	61	59	31	32	122
5 ≤ years <6	43	15	13	71	67	27	27	121
6 ≤ years <7	26	16	15	57	66	36	38	140
7 ≤ years <8	40	15	23	78	69	34	28	131
8 ≤ years <9	45	23	24	92	60	39	25	124
9 ≤ years <10	55	27	24	106	69	28	24	121
10 ≤ years <11	45	16	18	79	62	26	23	111
11 ≤ years <12	47	17	12	76	67	34	35	136
12 ≤ years <13	44	31	36	111	58	41	32	131
13 ≤ years <14	42	28	13	83	67	27	39	133
14 ≤ years <15	47	23	26	96	65	17	28	110
15 ≤ years <16	50	27	20	97	45	22	21	88
16 ≤ years <17	27	6	10	43	25	10	13	48
17 ≤ years <18	13	2	6	21	14	5	7	26
18 ≤ years <19	2	1	2	5	1	0	1	2
19 ≤ years <20	0	0	0	0	0	0	0	0
Total	619	296	291	1206	1015*	522	517	2054

MMS=multimodal screening. USS=ultrasound screening. *In 1 additional case date of diagnosis was not available.

Web Table 5: Sensitivity analysis for primary analysis

Methods

Sensitivity analyses were undertaken for three particular issues 1) (non-)equivalence of data source 2) correlation of data within regional centres and 3) competing deaths.

1) Data-source: There was concern that there could be more comprehensive data in the MMS and USS groups than the no screening group, given the potential for more sources of information for these women. This analysis only considered data obtained or triggered from the receipt of electronic health records (eHR), where equivalence between groups could be guaranteed. This meant that not only data pertaining to OC death, but also any data pertaining to any reason for early censorship, from eHRs was used to create the necessary failure-event and time variables employed by the software. The primary analysis utilizing the Versatile test was repeated on data created according to the described criteria.

2) Correlation of data: Data may be correlated within centres. This could be due to systematic differences in delivery of the screen at the 13 centres or also due to regional socio-economic differences leading to variability in OC incidence and mortality outcome. The Versatile test cannot be modified to account for this. However, the standard Cox analysis on the primary outcome was a) fitted with the standard errors calculated using a 'cluster robust' sandwich estimator [Stata command `stcox` with model option `vce(cluster` *`varname`*`)`], and the effect on standard errors and significance was observed. In addition, b) a random effects flexible parametric model [Stata command `mestreg` or `stmixed`] was also be fitted to assess the effect a random intercept term for centre had on the mortality reduction estimate and standard error. Furthermore, this model allowed testing of the random effect variance, and hence the level of unexplained variability between centres.

3) Competing deaths: There is a possibility that the effect of 'competing risks' may impact on the primary outcome. These competing risk events may be death from another cause when the woman had ovarian or tubal cancer, or death from a related cancer (for example, endometrial or breast) that may not be reasonably considered as uninformative censoring. A competing risks model [Stata command `stcrreg`] incorporating the above events as competing risks was used to whether the Cox model primary outcome HR estimates were essentially no different to the sub-HRs from a competing risks model.

Web Table 5

1) Data source: eHR- equivalent only (Versatile test)		
	max χ^2	p-value
MMS vs no screening	0.54	0.596
USS vs no screening	1.18	0.374

2a) Regional Cenres - cluster robust SEs					
	HR	se	L95% CI	U95% CI	p-value
MMS vs no screening	0.956	0.051	0.860	1.062	0.398
USS vs no screening	0.938	0.069	0.812	1.083	0.380

2b) Regional Centres - mixed survival model*					
	HR	se	L95% CI	U95% CI	p-value
MMS vs no screening	0.956	0.068	0.832	1.098	0.524
USS vs no screening	0.938	0.067	0.816	1.078	0.365

3) Competing risks - other deaths					
	HR**	se	L95% CI	U95% CI	p-value
MMS vs no screening	0.955	0.067	0.832	1.097	0.515
USS vs no screening	0.939	0.067	0.817	1.080	0.378

eHR=electronic health record. MMS=multimodal screening. USS=ultrasound screening. max $\chi2$ = maximum chi-squared values from the 3 weighted log-rank tests. HR=hazard ratio. SE/se=standard error. L95% CI=lower 95% confidence interval bound. U95% CI=upper 95% confidence interval bound. *test of random effect for RC: p=0.039 ** technically a sub-hazard-ratio

Web Table 6: Royston-Parmar model based estimates of the effect of screening at the pre-specified time-points of 5, 10, 15 and 18 years since randomization

A: No of deaths avoided (absolute survival difference) per 100 000 women						
Time-point (years)	MMS vs no screening			USS vs no screening		
	Estimate	L95% CI	U95% CI	Estimate	L95% CI	U95% CI
5	-0.9	-28.2	26.5	-3	-30.7	24.6
10	6.6	-43	56.1	7.1	-42.4	56.6
15	22.8	-53.1	98.8	31.6	-44	107.1
18	36.7	-65.3	138.8	52.9	-48.2	153.9
**B: Hazard ratio**						
Time-point (years)	MMS vs no screening			USS vs no screening		
	Estimate	L95% CI	U95% CI	Estimate	L95% CI	U95% CI
5	0.98	0.82	1.18	0.98	0.82	1.17
10	0.95	0.82	1.09	0.93	0.8	1.07
15	0.93	0.78	1.12	0.9	0.75	1.08
18	0.92	0.75	1.14	0.88	0.72	1.09

MMS=multimodal screening. USS=ultrasound screening. CI=Confidence Interval

C. Supplementary Figures

Web Figure 1: Cumulative invasive epithelial ovarian and tubal cancer deaths

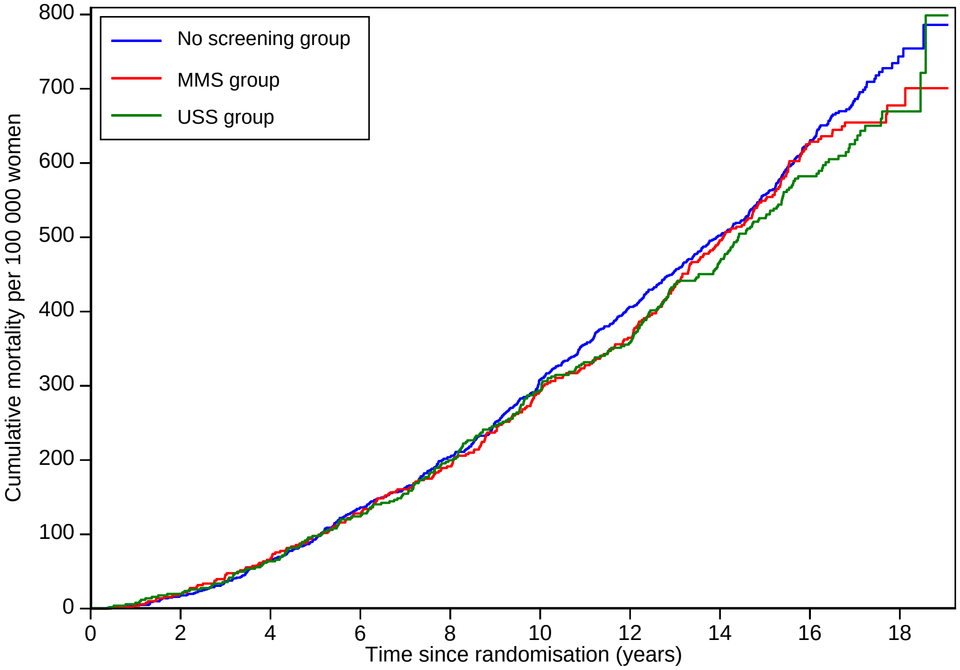

Numbers at risk

No screening	101314	(18)	100761	(47)	99751	(71)	98393	(66)	96854	(100)	94251	(91)	90830	(86)	87495	(96)	58093	(40)	10333
MMS	50625	(10)	50359	(23)	49883	(31)	49235	(31)	48429	(49)	47173	(33)	45529	(59)	43862	(50)	29014	(8)	5193
USS	50622	(10)	50351	(22)	49862	(30)	49247	(37)	48451	(45)	47199	(30)	45635	(49)	43994	(46)	29165	(16)	5255

Web Figure 2a: Cumulative ovarian and tubal cancer deaths by randomisation group with RP model fit overlaid - MMS versus no screening

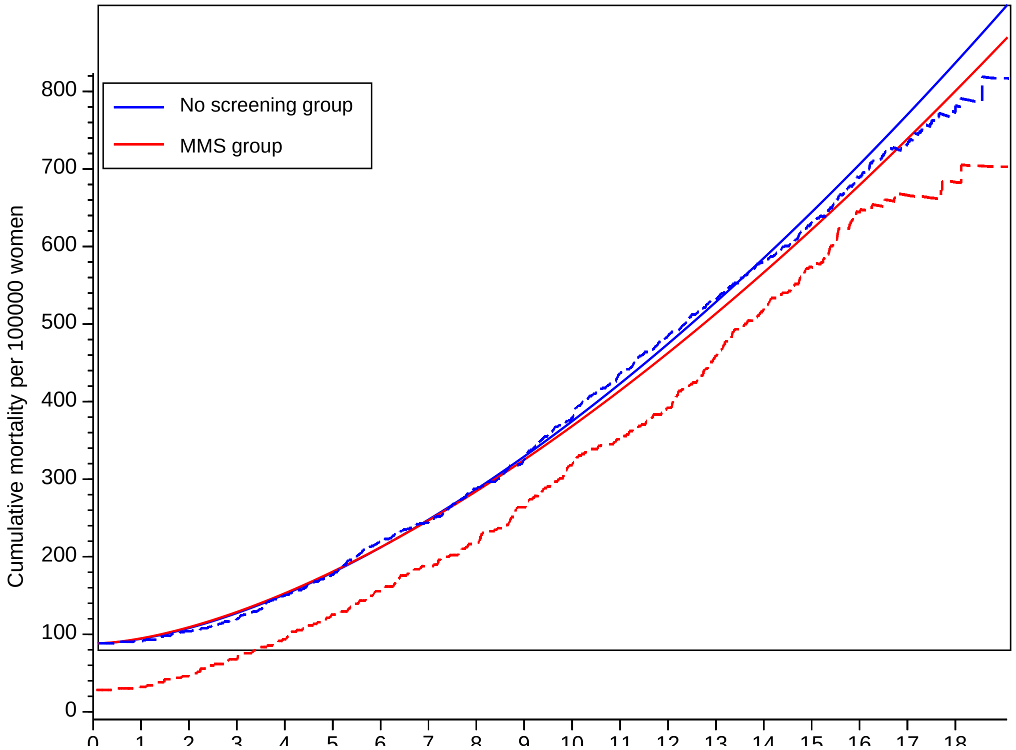

Web Figure 2b: Cumulative ovarian and tubal cancer deaths by randomisation group with RP model fit overlaid - USS versus no screening

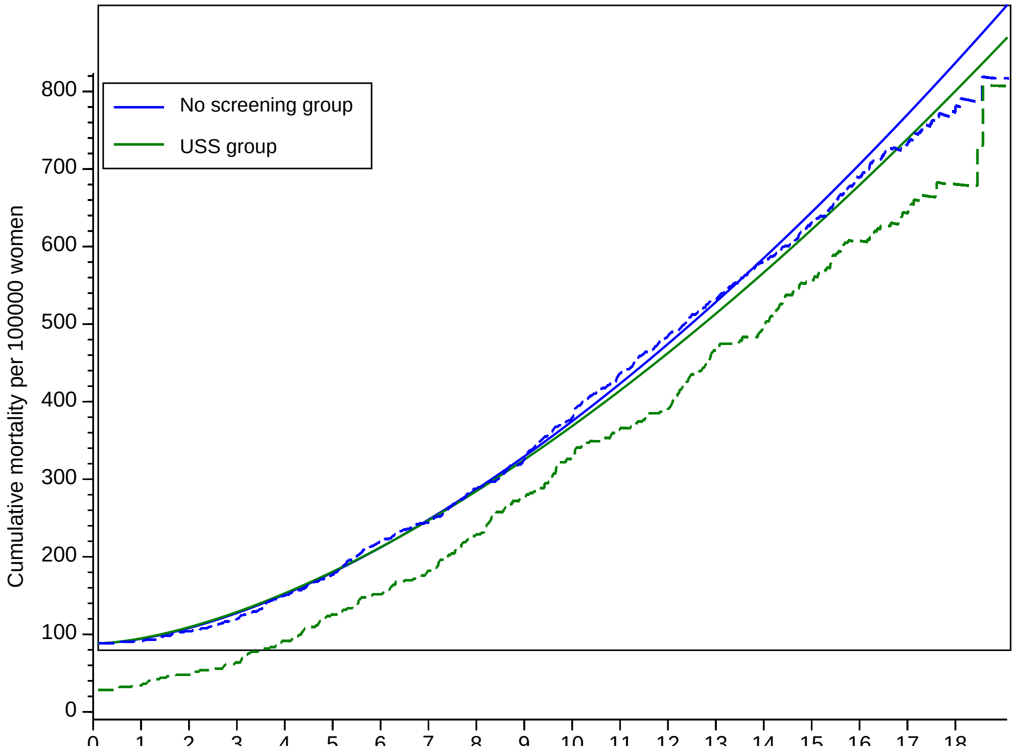

Web Figure 3a: RP model estimate of ovarian and tubal cancer death rates (hazard function) with 95% confidence limits – MMS versus no screening

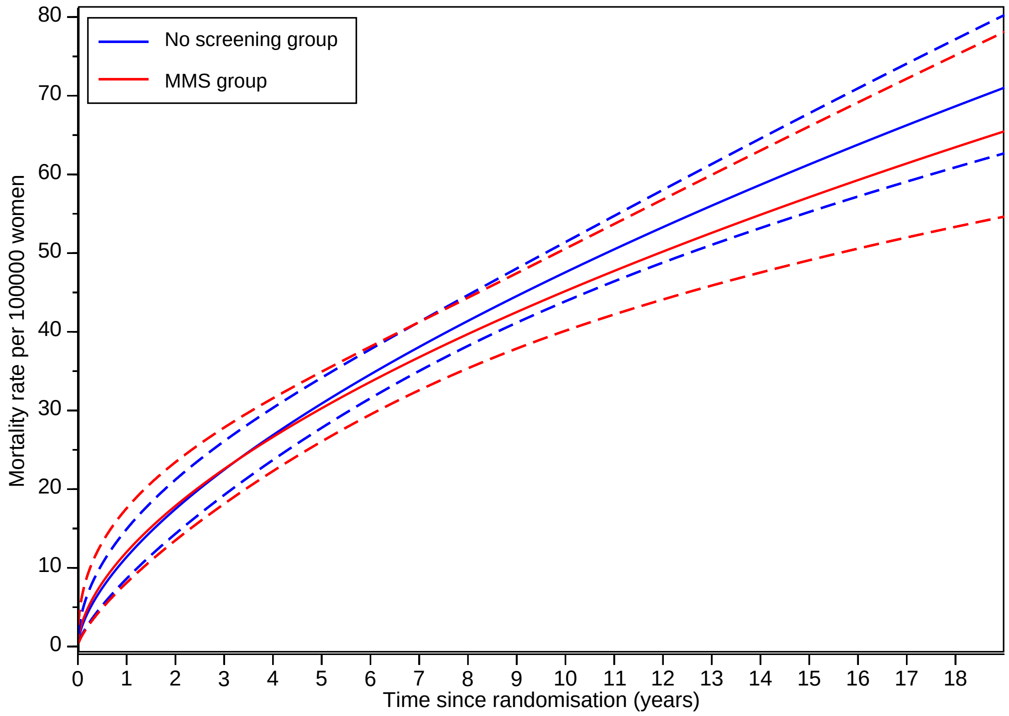

Web Figure 3b: RP model estimate of ovarian and tubal cancer death rates (hazard function) with 95% confidence limits – USS versus no screening

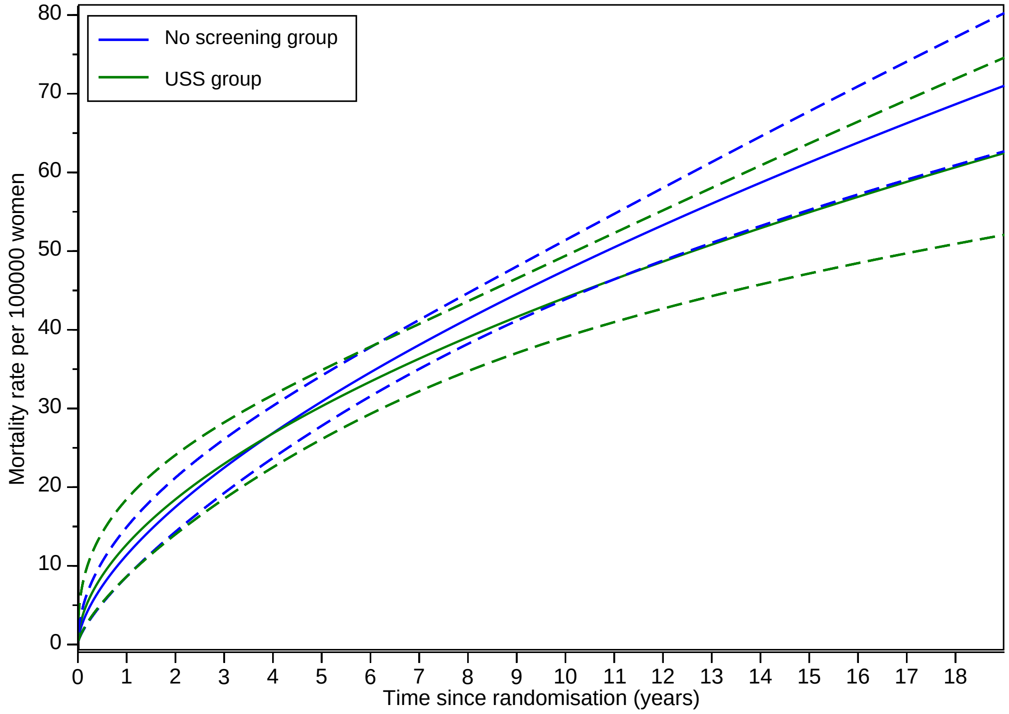

Web Figure 4a: RP model estimate of ovarian and tubal cancer death hazard ratio for MMS versus no screening with 95% confidence limits

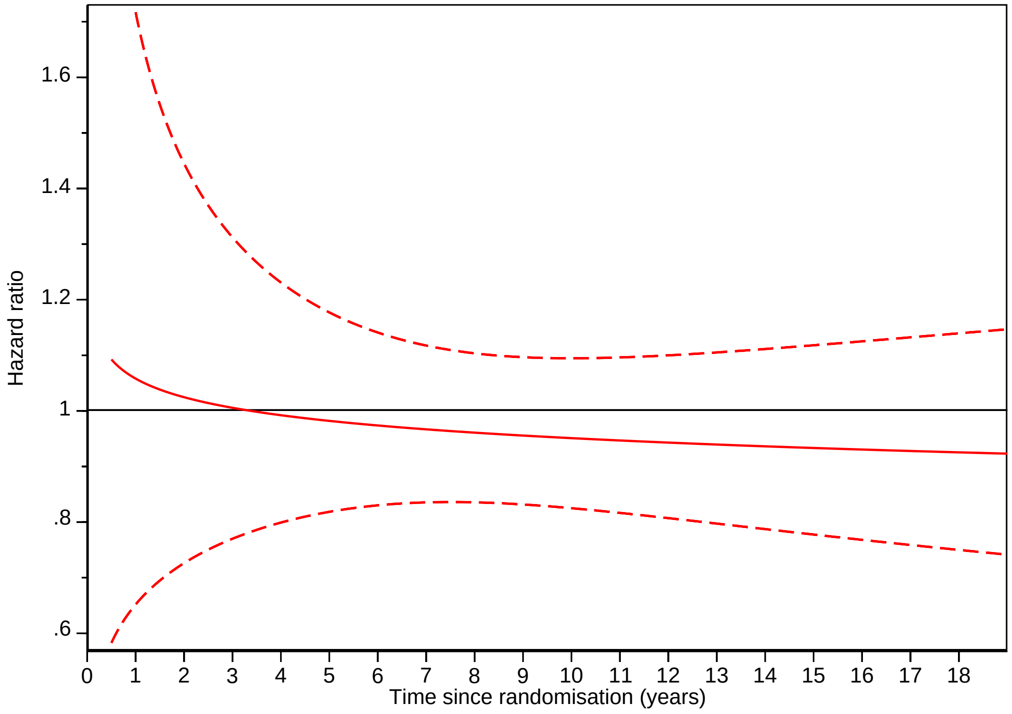

Web Figure 4b: RP model estimate of ovarian and tubal cancer death hazard ratio for USS versus no screening with 95% confidence limits

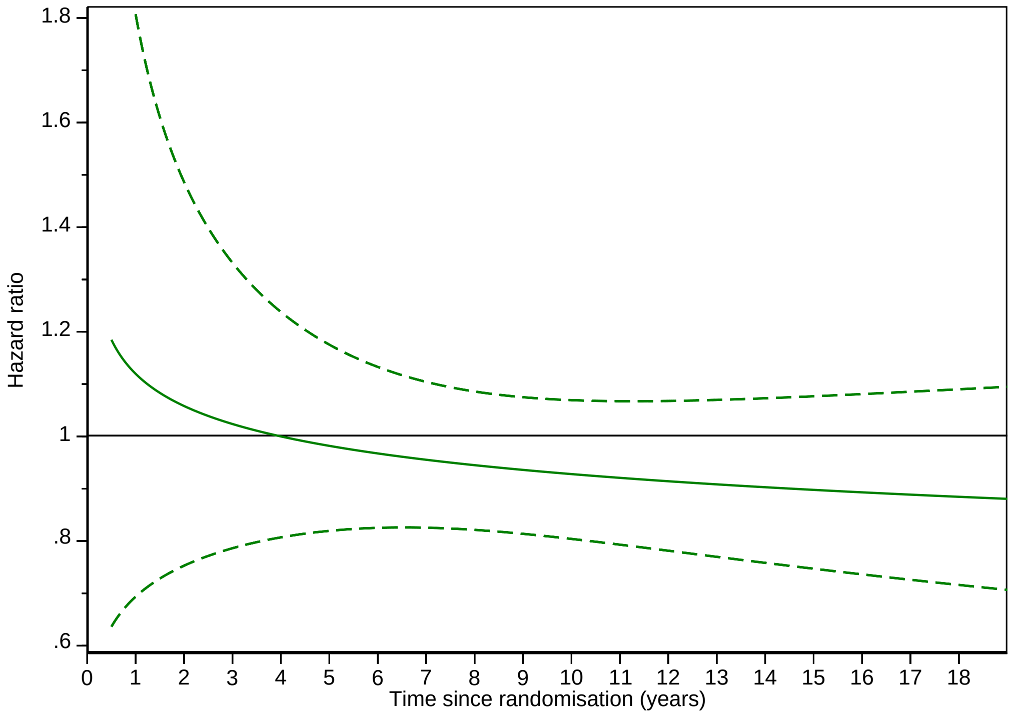

Web Figure 5: Survival rates in women with ovarian and tubal cancer in the no screening group compared with one-, and five- year age and period adjusted survival rates in the UK population. 10 year survival rates by age group not available for the UK population.

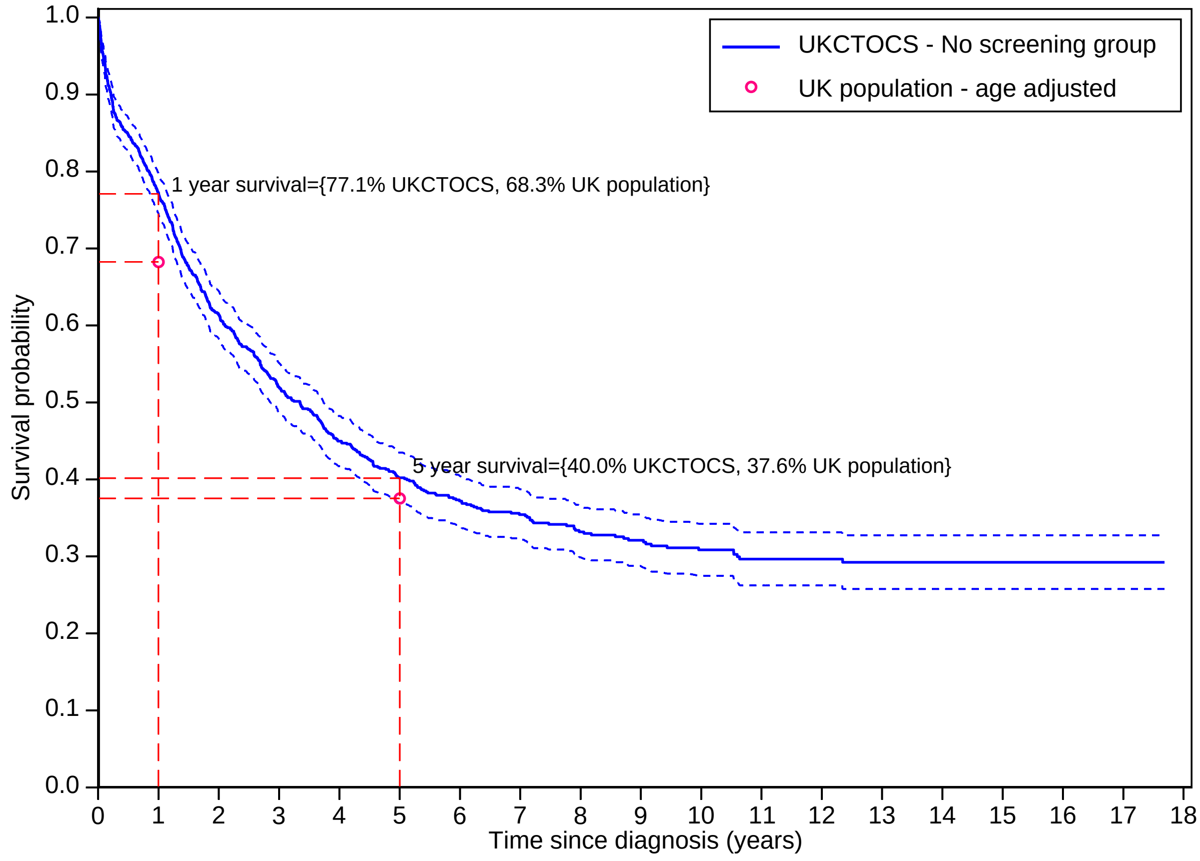

Web Figure 6a: Cumulative incidence rates (hazard functions) of ovarian and tubal cancer with 95% confidence bands MMS versus no screening

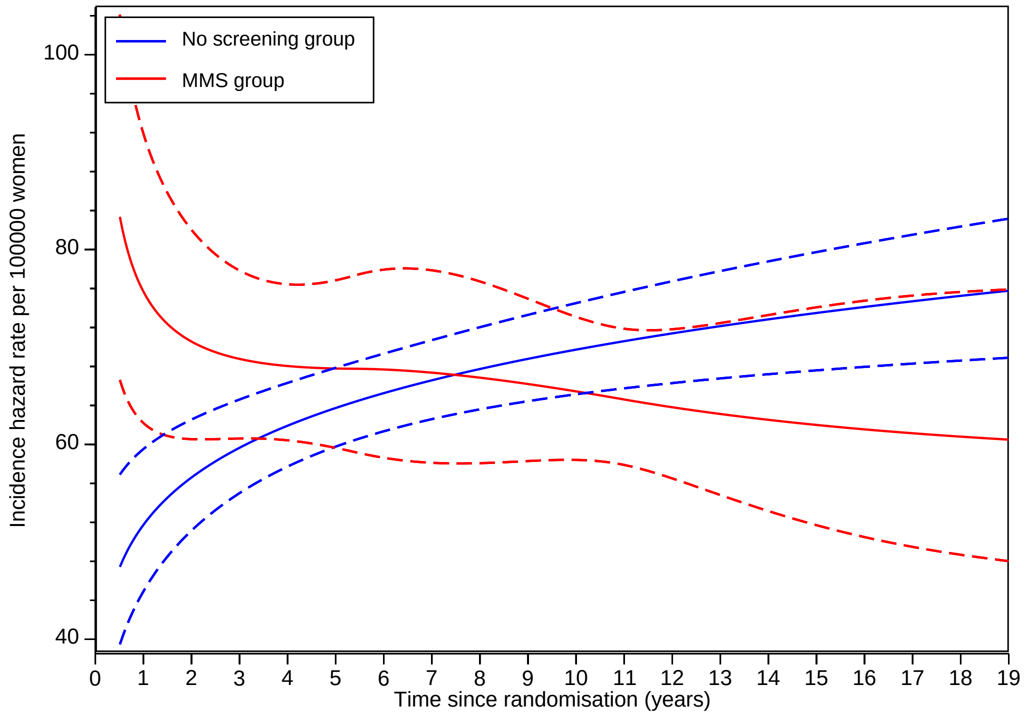

Web Figure 6b: Cumulative incidence rates (hazard functions) of ovarian and tubal cancer with 95% confidence bands USS versus no screening

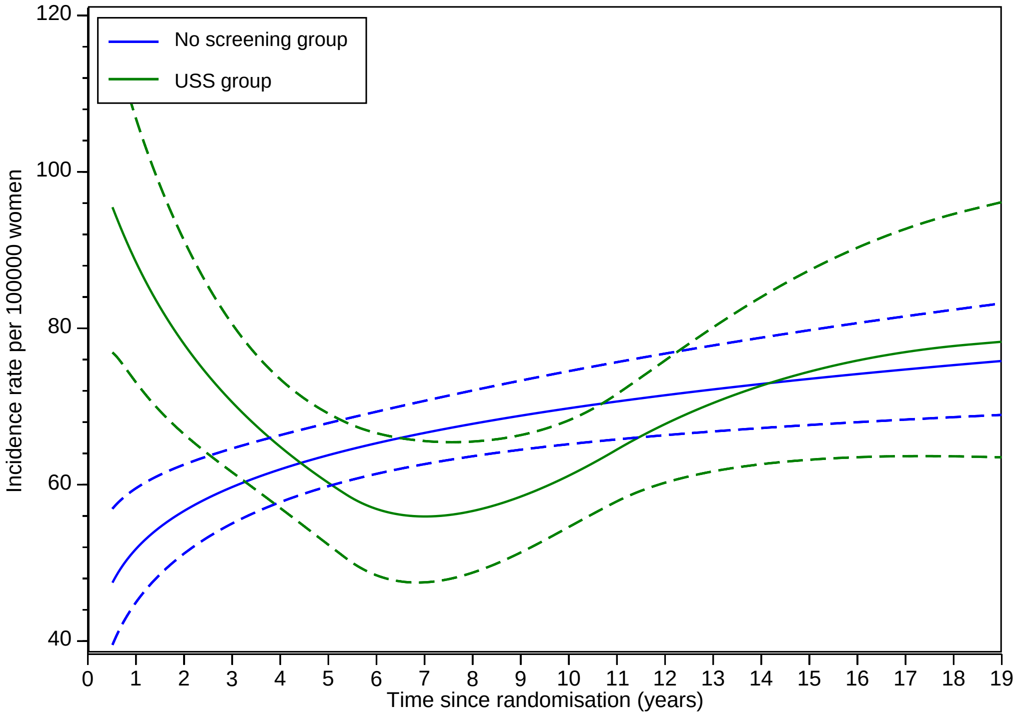

D. Long term follow up of UKCTOCS committees and teams

Trial Steering Committee

Prof H Kitchener (Chair; independent member), Prof Dame J Patnick (independent member), Ms A Jones (independent member & lay representative), Prof J Cuzick (independent member), Prof U Menon, Prof I Jacobs, Prof M Parmar, Prof Dame L Fallowfield.

Trial Management Committee

Core: Prof U Menon (Chair), Prof I Jacobs, Prof M Parmar, Dr S Skates, Prof S Campbell, Prof A McGuire, Prof Dame Lesley Fallowfield, Mr R Woolas, Prof N Singh, Dr A Gentry-Maharaj, Dr A Ryan, Dr M Burnell, Dr J Kalsi.

Extended: Dr A Dawnay, Mr T Mould, Mr MW Seif, Ms A Sharma, Ms K Williamson, Mr S Leeson, Mr S Dobbs

Outcome Review Committee

Prof N Singh (Chair), Mr R Woolas, Prof R Manchandra, Ms K Williamson, Ms A Sharma, Dr R Arora, Dr L Casey.

Trial Management Team

Prof U Menon (Chair), Dr A Gentry-Maharaj (Project Lead), Dr A Ryan, Dr M Burnell, Ms G Carlino, Ms C Karpinskyj, Mrs S Massingham, Ms R Payne, Mrs A Widdup.
